# Can implementation of a complex outpatient antimicrobial therapy program reduce readmissions for patients with bone and joint infections?

**DOI:** 10.1017/ash.2025.10274

**Published:** 2026-01-26

**Authors:** Elizabeth Thottacherry, Marten Hawkins, Jallisae Nedi, Susan Ellen Turley, Patrick Facelo, Timothy Pierce, Noah Fang, Daisuke Furukawa

**Affiliations:** 1 Division of Infectious Diseases and Geographic Medicine, Stanford University School of Medicine, Stanford, CA, USA; 2 Division of Infectious Diseases, University of Michigan Medical School, Ann Arbor, MI, USA; 3 Stanford Healthcare, Stanford, CA, USA; 4 Hospital Based Practices, Indiana University Health, Indianapolis, IN, USA

## Abstract

**Objective:**

Evaluate whether a complex outpatient antimicrobial therapy (COpAT) program led by advanced practice providers (APPs) conducting transition-of-care (TOC) services improves post discharge patient follow-up and reduces hospital readmission.

**Design:**

Pre- and postimplementation cohort study comparing outcomes 6 months before and 5 months after COpAT launch.

**Setting:**

706-bed tertiary university teaching hospital.

**Patients:**

Adult patients admitted to the hospital with bone and joint infections, seen by our inpatient infectious disease consultation service and discharged with at least fourteen days of antimicrobial therapy, with follow-up at our specialized musculoskeletal infectious diseases clinic.

**Intervention:**

The APP led pilot COpAT program was launched on March 1^st^, 2024, with a multidisciplinary team including an infectious diseases physician, APP, nurse, medical assistant, and TOC pharmacists. Patients enrolled at hospital discharge and were scheduled for APP-led TOC visits within fourteen days, followed by a physician visit.

**Results:**

The pre- and post-intervention groups included 100 and 135 patients, respectively. Mean postdischarge follow-up time decreased from 20.1 to 9.1 days (*P* < .001), and patients seen within fourteen days increased from 42% to 83% (*P* < .001). Readmission rates and emergency room visits did not change significantly. TOC pharmacy engagement rose from 8% to 42% (*P* < .001), and both TOC pharmacy and APP interventions frequently addressed medication errors, side effects, and treatment modifications.

**Conclusion:**

A structured, COpAT program with APP and TOC pharmacy involvement optimizes postdischarge follow-up, strengthens antimicrobial outpatient monitoring, and supports timely intervention for patients with complex infections.

## Introduction

Outpatient parenteral antimicrobial therapy (OPAT) has been widely used since the 1970s to manage invasive infections in outpatient settings, such as patient’s homes, infusion centers, or skilled nursing facilities.^1^ OPAT programs require multidisciplinary input and have demonstrated several benefits including multibillion-dollar savings through reduced hospital stays, lower rates of healthcare-associated infections as well as greater patient satisfaction.^2,3^ Building on these foundational principles of OPAT, complex outpatient antimicrobial therapy (COpAT) programs expand outpatient management by incorporating both oral and intravenous antimicrobial therapy. COpAT programs are not well defined, with historical iterations having a focus on intravenous antimicrobial therapy in the community.^4^ Current COpAT programs reflect evolving trends favoring the use of oral antimicrobials for conditions such as bone and joint infections and endocarditis, and COpAT programs are increasingly utilized to monitor patients on prolonged or high risk.^5–9^ While the use of COpAT reflects evolving treatment trends, data on its management, and outcomes remain scarce.^7^

COpAT providers face many challenges, including monitoring prolonged courses of antimicrobial therapy and managing adverse effects.^5,7,10^ While clinical pharmacists assist with lab monitoring and dose adjustments, the responsibility for managing antimicrobial changes, and side effects often falls to the infectious diseases physician.^7,11,12^ One strategy to address the uncompensated burden of care involves the utilization of transition-of-care (TOC) visits, which are postdischarge clinic visits performed by physicians or advanced practice providers (APPs) focused on continuity of care, medication management, and early detection of complications.^13–15^ However, their role in COpAT programs has not been studied, and their impact on patient outcomes in this context remains unclear.

In this study, we evaluated the impact of a pilot APP-driven COpAT program at our institution. We hypothesized that a COpAT program with an APP who could perform postdischarge TOC visits would allow faster access to care postdischarge for patients on prolonged antimicrobial therapy, thereby improving outpatient monitoring and reducing complications as well as adverse events.

## Methods

### Patient population and setting

The criteria for inclusion into the pilot COpAT program were 1) adult patients admitted to our hospital with a bone and joint infection, 2) seen by our inpatient infectious diseases consultation service, 3) discharged with at least fourteen days of antimicrobial therapy, and 4) follow-up scheduled at our specialized musculoskeletal infectious diseases clinic. Our hospital is a 706-bed tertiary university teaching hospital with five inpatient infectious diseases consult services based on admitting team’s specialty. Patients with bone and joint infections were predominantly seen by the surgical-infectious diseases service if admitted by a surgical specialty or the medical-infectious diseases service if admitted by a medical specialty. Discharge antibiotic plans were created by the inpatient infectious diseases consult service with infectious diseases clinical pharmacists providing support as needed. Our musculoskeletal infectious diseases clinic is a specialized clinic where patients are co-managed by infectious diseases physicians and orthopedic surgeons or wound care specialists.

### COpAT program

The pilot COpAT program comprised of one nurse, one medical assistant, one APP, and one physician medical director. The nurse coordinated care by facilitating postdischarge follow-up appointments and communicating with nursing homes and home health agencies. The medical assistant was responsible for scheduling follow-up appointments and obtaining surveillance lab test results for patients on intravenous and select oral antimicrobials. A nurse practitioner was hired as an APP prior to program initiation, with the ability to perform both in person and telehealth clinic visits. They primarily conducted TOC postdischarge visits as telehealth visits as described below and acted as the primary point of contact for COpAT related patient care concerns, including review of surveillance labs. Lastly the physician medical director provided program oversight and addressed patient care issues as needed.

The COpAT program also piloted a collaboration with the inpatient TOC pharmacy service. This consult service, previously available only to Internal Medicine admitting teams, provides medication review and discharge counseling for patients with high-risk medical conditions, such as heart failure or myocardial infarctions, or those on high-risk medications such as anticoagulants, antiarrhythmics, or antimicrobials. Pharmacists performed a comprehensive medication review including assessment of current labs, comorbidities, and drug–drug interactions and relayed recommendations to the ordering provider. To prevent interruptions in therapy, the pharmacists also confirmed discharge medication access by ensuring the medication was in stock, covered by insurance, and ready for pick-up at the patients’ preferred pharmacy.

### Interventions

The COpAT program was formally implemented March 1^st^ 2024. On this date, the following interventions were implemented: expansion of the inpatient TOC pharmacy service and implementation of the TOC postdischarge visit. These interventions were aimed at improving patient care during the transitional period between inpatient and outpatient setting.

1. *TOC pharmacy expansion*

The eligibility for TOC pharmacy consult was expanded to include patients who were admitted to a non-Medicine primary team (eg, orthopedic surgery) who were discharged with long-term oral antimicrobials. Patients on intravenous antimicrobials were excluded from this expansion effort because these patients often received additional counseling from their home health agencies.

1. *Postdischarge TOC visits*

A new workflow was implemented for the APP to conduct a TOC visit with the patient within fourteen days of discharge. When eligible, visits were conducted as a Transition of Care Management Service, a Medicare-billable service supporting patients transitioning back to the community setting post hospital discharge. Eligible patients had documented or attempted postdischarge communication within 48 hours and scheduled clinic follow-up within fourteen days to fulfil Transition of Care Management Service requirements. Current Procedural Terminology code 99496 was used for patients seen within seven days of discharge and code 99495 for those seen within fourteen days.^14^ The APP was specifically trained to review and help carry out the antibiotic plan created by the infectious diseases consult service post discharge. They reviewed antimicrobial adherence and surveillance lab results, provided medication counseling, and addressed side effects. These visits did not replace regularly scheduled follow-up visits with the physician, and management decisions such as defining antimicrobial end of therapy were deferred to the physician visit. In addition to implementing the TOC visit, efforts were also made to optimize the post discharge scheduling workflow to decrease the time to physician visit post discharge.

### Data collection and analysis

This study was a pre vs postimplementation cohort study evaluating the impact of the COpAT program. Both pre- and postimplementation cohorts were identified retrospectively from our internal bone and joint infection database. Both cohorts were reviewed to ensure they met inclusion criteria for the COpAT program via manual chart review. The preimplementation cohort included patients who were discharged from September 1^st^, 2023, to February 28^th^, 2024, and the postimplementation cohort included patients who were discharged from March 1^st^, 2024, to July 31^st^, 2024. Patient data were collected via a data pull conducted by our informaticist team and supplemented by additional manual chart review. The postimplementation period was limited to five months because the study was initiated at that point, and data through that time was available for review, allowing timely analysis and planning for expansion to the broader infectious diseases clinic.

Results were first described descriptively. Mean and standard deviation were used for continuous variables, while proportion and frequencies were used for categorical variables. For group comparisons, *T*-test was used for continuous variables, and χ^2^ test was used for categorical variables. We used STATA version 16 software (STATA Corp, College Station, TX) for all analyses. The institutional review board at our institution approved this study.

## Results

Overall 100 patients were included in the preintervention population and 135 were included in the post-intervention population. Patients had a wide variety of bone and joint infections including native bone and joint infections and hardware-associated infections, with similar distributions observed in both the pre- and post-intervention arms. An exception to this was patients with diabetic foot osteomyelitis, as a significantly larger proportion were included in the post-intervention arm. Given the small number of patients, it is difficult to draw conclusions about how this discrepancy may have influenced results. A significant proportion of our patients were enrolled in Medicare, with the remainder insured under MediCal, California’s state Medicaid insurance or private insurance. Most patients were discharged exclusively on oral antimicrobials in both cohorts. Close to 60% of patients were discharged home from the hospital, while the rest were discharged to either skilled nursing facilities or acute rehab facilities. These demographics are displayed in Table 1.

**Table 1.** Baseline demographic information of the pre- and post-intervention cohorts

	Pre-intervention N = 100 (%)	Post-intervention N = 135 (%)	*P*-value
**Mean age**	64.5 (1.6)	65.2 (1.3)	.74
**Male**	60 (60)	88 (65)	.42
**Race**			.85
Hispanic	20 (20)	35 (26)	
Non-Hispanic White	56 (56)	67 (50)	
Non-Hispanic Black	6 (6)	8 (6)	
Non-Hispanic Asian	10 (10)	13 (10)	
Other/unknown	8 (8)	12 (9)	
**Insurance**			.6
Medicare	60 (60)	81 (60)	
Medical	24 (24)	34 (25)	
Private	13 (13)	19 (14)	
Other	3 (3)	1 (1)	
**Admitting team**			.64
Surgical	76 (76)	99 (73)	
Medical	24 (24)	36 (27)	
**Infection type**			
Prosthetic joint infection	39 (39)	40 (30)	.13
Hardware-associated infection	10 (10)	15 (11)	.79
Spondylodiskitis	15 915)	16 (12)	.48
Native osteomyelitis and septic joint	24 (24)	28 (21)	.55
Decubitus ulcer osteomyelitis	1 (1)	4 (3)	.3
Diabetic foot osteomyelitis	10 (10)	27 (27)	.04
Other	3 (3)	6 (4)	.57
**Discharge antimicrobial**			
Oral beta lactam	35 (35)	36 (27)	.17
Intravenous beta lactam	26 (26)	29 (21)	.41
Intravenous vancomycin	14 (14)	16 (12)	.63
Trimethoprim-Sulfamethoxazole	7 (7)	21 (16)	.05
Doxycycline	26 (26)	33 (24)	.79
Fluoroquinolones	15 (15)	25 (19)	.48
Antifungals	2 (2)	4 (3)	.64
Daptomycin	3 (3)	4 (3)	.99
Rifampin	7 (7)	9 (7)	.92
Other	3 (3)	7 (5)	.41
**Oral antimicrobials only**	62 (62)	92 (68)	.33
**Length of stay**	9.2 (.7)	9.6 (.7)	.74
**Discharge destination**			.73
Home	57 (57)	80 (59)	
Facility	43 (43)	55 (41)	

**Figure 1.**
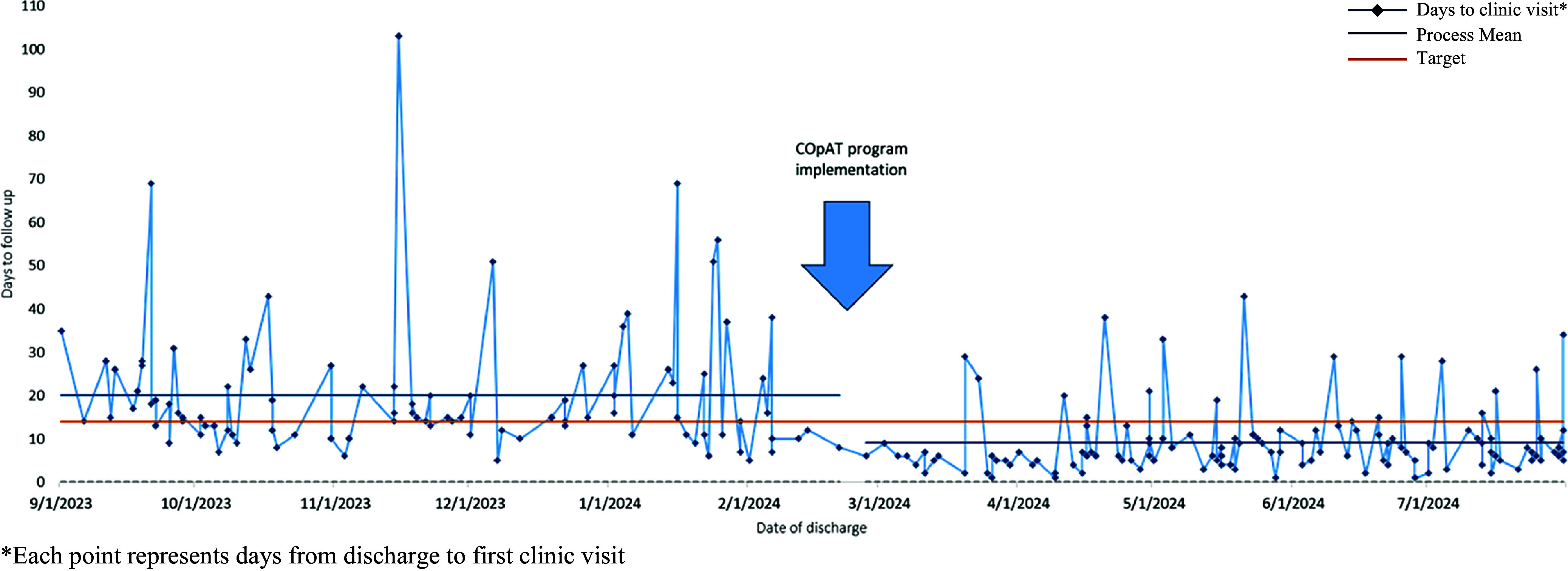
Days to first APP or physician visit.

Figure 1 depicts changes in number of days to first clinic follow-up post discharge. The highlighted target line represents fourteen days to clinic follow-up in accordance with billing requirements for a Transition of Care Management Service. The mean follow-up time with either physician or APP decreased from 20.1 to 9.7 days (*P* < .001). The percentage of patients seen by either physician or APP within 2 weeks of discharge increased from 42% (42/100) to 83% (112/135) (*P* < .001) post intervention. On closer examination, mean follow-up with physicians also decreased from 20.1 to 14.4 days (*P* < .001). The APP scheduled follow-up for 90 patients overall, with 76 patients billed as a Transition of Care Management Service per Medicare.

Utilization of TOC pharmacy at discharge increased from 8% (8/100) to 42% (57/135) (*P* < .001). Table 2 summarizes the interventions performed by the TOC pharmacy during the post intervention period. Medication review and patient/care giver education were the most common interventions. Counseling points emphasized include separating divalent supplements with certain antimicrobials or emphasizing certain block-box warnings or side effects. Some notable examples of medication access interventions included transferring an antimicrobial prescription to another local pharmacy due to patient moving, obtaining a partial fill for an antimicrobial needed same day, and arranging an antimicrobial to be delivered to the hospital prior to discharge. Direct clinical interventions included dose adjustments (eg, dose adjustment of trimethoprim-sulfamethoxazole based on weight and adjusting warfarin dose for drug interaction) as well as correcting duplicate or omitted medication orders.

**Table 2.** Summary of interventions performed by the transition of care inpatient pharmacy

Types of intervention	PostCohort, n = 59
Comprehensive medication review	55
Patient/caregiver education	52
Medication access resolved	10
Clinical interventions	
Dose adjustment	18
Medication omission	4
Medication duplication	5

Table 3 summarizes the interventions performed post discharge by the APP during the 5-month postintervention period, both at the APP-led TOC visit and prior to physician follow-up. Ordering missing surveillance lab tests was the most common intervention performed by the APP. Other frequent interventions included managing adverse events such as medication side effects, lab abnormalities, and referring patients to the emergency room for acute issues. Examples included discontinuing trimethoprim-sulfamethoxazole due to hyperkalemia and acute kidney injury, and dose adjustment of intravenous vancomycin based on renal function and trough levels. Symptom management interventions included providing counseling on drug administration and prescribing anti-nausea medication to minimize gastrointestinal intolerance, as well as prescribing antihistamines and recommending drug cessation in response to new onset rash.

**Table 3.** Summary of interventions performed by the advanced practice provider

Types of intervention*	Post cohort, n = 135
Ordering missing labs	21
Lab abnormalities addressed	17
Adverse event leading to modification in therapy	12
Medication counseling	8
Medication error addressed	6
Sent to emergency room	5
Prescription for symptoms	3
Notified surgery for orthopedic issue	3
Other**	4

* These interventions are not mutually exclusive. In some instances, multiple interventions were performed during the same encounter.

** Other: placing antimicrobial continuation order, ordering electrocardiogram, ordering central catheter line care, ordering interventional radiology referral for central catheter line removal.

There was no difference in all cause 30-day readmission rates between the pre- and post-intervention cohorts (15% [15/100] pre vs 11% [15/135] post) (*P* = .377) and in 30-day emergency room visits (10% [10/100] pre vs 15% [20/135] post) (*P* = .234).

## Discussion

Our study demonstrated that a multidisciplinary COpAT team involving a TOC pharmacy team and an APP can improve time to outpatient follow-up and support clinical decision-making prior to and postdischarge. By implementing TOC visits, we significantly reduced the time to first patient contact post discharge and increased the proportion of patients seen within fourteen days of discharge.

We note that faster follow-up did not demonstrate a significant change in readmissions or emergency room visits. This may be due to earlier identification of medication related adverse events, resulting in appropriate patient referrals to the emergency room. Additionally, unlike past studies of OPAT programs which strictly evaluated patients on intravenous antimicrobials, most of our patients were discharged on oral antimicrobials, which have lower rates of serious adverse effects.^5,6,8,9^ As such, our impact on reducing readmissions and emergency room visits may have been minimized. Similarly, the TOC pharmacy interventions were limited to patients on oral antimicrobials and might have had a greater effect had patients on intravenous antimicrobials been included.

Despite the overall safety of oral compared to intravenous antimicrobials, long-term antimicrobial therapy still carries significant medication risks, side effects, and errors requiring close outpatient monitoring.^10,13,16,17^ The novel inclusion of both inpatient TOC pharmacists and a dedicated COpAT APP added an additional layer of antimicrobial medication review, patient education, and side effect management. Our institution does not have infectious diseases clinical pharmacists routinely reviewing discharge antibiotic plans, so TOC pharmacists therefore played a critical role in addressing this gap, demonstrating that this model is feasible and replicable in hospitals without dedicated infectious diseases pharmacy services. The APP aided antimicrobial monitoring immediately after discharge, identifying and managing predictable gaps in care prior to physician review as shown in Table 3. Traditionally, tasks such as ordering surveillance lab tests, managing post discharge symptoms, and addressing lab abnormalities fall to inpatient teams and outpatient infectious disease physician after a patient is discharged. The involvement of our APP helped redistribute this responsibility and reinforced overall post discharge safety. The frequency of interventions performed by our APP and TOC pharmacists underscores the need for close monitoring of patients on long-term antimicrobial therapy. Prior studies show adverse side effects frequently occur early in the OPAT treatment course.^18^ Because all patients included in the pre- and post-intervention arms received antimicrobial therapy for greater than fourteen days, early identification in medication errors and side effects prior to infectious diseases physician visit was instrumental in preventing adverse patient outcomes.

We do note some limitations in our study. First, our COpAT program was limited to our musculoskeletal infectious disease clinic at the time of program initiation and so data collected may not be widely applicable to patients with other infections. Second, we acknowledge that all patients included in our program were insured and had stable housing on discharge (either to a facility or home) and were able to participate in post discharge clinic visits. Our program may be less impactful in a healthcare setting where reliable follow-up cannot be ensured. Third, given the period in which the two cohorts were selected, there may have been seasonal variations in patient characteristics and clinic operations that were unaccounted for in our analysis. Fourth, though we demonstrated a numeric decrease in readmission rates, our study was underpowered to assess true differences in rates of emergency room visits and hospital readmissions. Lastly, comorbidities were not captured in our study, which may have impacted emergency room visits and readmission rates. Nevertheless, by demonstrating earlier follow-up and detailing the breadth of interventions performed, we highlighted the meaningful clinical impact our COpAT program had on patient care.

Looking forward, there continues to be ample opportunity to streamline antimicrobial administration and monitoring in the outpatient setting within the COpAT program. Enhancing the electronic medical record system to automate tasks like antimicrobial orders and lab result retrieval could reduce human error. Employing artificial intelligence has the potential to revolutionize COpAT programs in the future, possibly reducing the need for manual review of data and predicting future medication-related adverse events.^5^

To conclude, a COpAT program with involvement of a TOC inpatient pharmacy team and TOC APP visits can effectively bridge the gap between hospital discharge to outpatient infectious disease physician follow-up. This model increases medication monitoring, enhances patient education, and facilitates early detection and management of adverse events. As oral antimicrobial use continues to expand for the treatment of serious infections, a structured COpAT program such as ours is critical in optimizing patient care.

## Prior presentations

This work was presented at the 2025 International Forum on Quality and Safety in Healthcare, Royal Jaarbeurs, Utrecht, on May 23 2025, highlighting quality improvement strategies in outpatient antimicrobial monitoring.
